# Dual-task gait assessment using n-back testing during self-paced treadmill walking in virtual reality: reliability and baseline characterisation for future clinical application

**DOI:** 10.3389/fnhum.2026.1807769

**Published:** 2026-07-01

**Authors:** Mohammad Al-Amri, Rula Abdallat, Abdulrhman Mashabi, Hilal Al Balushi

**Affiliations:** 1 School of Healthcare Sciences, Cardiff University, Cardiff, United Kingdom; 2 Department of Biomedical Engineering, Faculty of Engineering, The Hashemite University, Zarqa, Jordan; 3 Department of Physical Therapy, College of Medical Rehabilitation Sciences, Taibah University, Madinah, Saudi Arabia; 4 Physiotherapy and Rehabilitation Department, Sultan Qaboos University Hospital, University Medical City, Seeb, Oman

**Keywords:** dual-tasking walking, gait stability, self-paced treadmill, virtual reality treadmill, working memory

## Abstract

**Background:**

Dual-task gait paradigms are widely used to assess cognitive–motor integration, however considerable methodological heterogeneity remains. Fixed-speed locomotion and limited assessment of repeatability have contributed to inconsistent baseline findings in healthy adults, complicating interpretation of dual-task effects reported in clinical populations.

**Objective:**

This study aimed to examine the reliability and baseline gait behaviour associated with a dual-task paradigm combining non-immersive virtual reality (VR)-based self-paced treadmill walking and parametrically modulated working memory load. The purpose was to establish a robust reference profile in healthy adults to support subsequent clinical and reliability and baseline characterisation studies.

**Methods:**

Twenty-three healthy adult males (34.56 ± 5.12 years) completed two gait assessment sessions using a self-paced treadmill within a non-immersive VR environment. Walking was assessed under three conditions: single-task walking, dual-task walking with a 1-back task, and dual-task walking with a 2-back task. Spatiotemporal gait parameters and dual-task costs were analysed across sessions and task conditions to evaluate between-session consistency and sensitivity to graded cognitive demand.

**Results:**

Most spatiotemporal gait parameters demonstrated good consistency between sessions, with no statistically significant differences observed across days or task conditions (*p* ≥ 0.05). A reduction in step length was observed during dual-task walking in the first session (*p* = 0.02), alongside consistent directional modulation of stride-related measures with increasing cognitive load. These changes were modest and consistent with adaptive gait regulation in neurologically intact adults.

**Conclusion:**

The findings demonstrate that a self-paced treadmill VR dual-task gait protocol yields stable and repeatable gait measures following familiarisation in healthy adults. Rather than establishing criterion validity, this study provides baseline characterisation and reliability evidence necessary for future work evaluating clinical sensitivity, diagnostic utility, and intervention responsiveness in patient populations.

## Introduction

Human locomotion in daily life is rarely a purely motor activity; rather, it is frequently performed alongside concurrent cognitive demands such as monitoring the environment, decision-making, or engaging working memory processes (Maiocchi et al., 2026; Smith et al., 2017). The ability to manage these simultaneous demands, commonly referred to as cognitive-motor integration, is fundamental to functional autonomy and safety, particularly in complex or unpredictable environments. Disruption of this integration is increasingly recognised as an early marker of neurological dysfunction, motivating the widespread use of dual-task paradigms in both research and clinical assessment (Nedović et al., 2025; Veldkamp et al., 2021). Crucially, understanding baseline dual-task behaviour in healthy adults is essential to generate normative data and provide a foundation for interpreting clinical findings.

Dual-task paradigms are commonly used to characterise dual-task interference, whereby the concurrent execution of motor and cognitive tasks leads to measurable changes in performance in one or both domains (Kelly et al., 2012; Huang and Mercer, 2001). Such interference may reveal subtle impairments not evident under single-task conditions and has been reported across a range of neurological populations, including Parkinson’s disease (Kelly et al., 2012), traumatic brain injury (Fino et al., 2018), and stroke (Cherry-Allen et al., 2018). Accordingly, dual-task gait assessment has gained traction as a clinically relevant approach for probing cognitive–motor dysfunction and monitoring recovery or disease progression.

A common implementation of dual-task gait assessment involves walking while performing a concurrent cognitive task such as the Stroop or N-back tests (Commandeur et al., 2018). Gait is typically recorded using overground instrumented walkways or fixed-speed treadmills, both of which allow controlled measurement of spatiotemporal parameters. However, these approaches impose methodological constraints: overground assessments are limited by space and trial duration, while fixed-speed treadmills constrain self-selected gait dynamics and may artificially alter stride characteristics and variability (Rohafza et al., 2022).

Self-paced treadmills address several of these limitations by allowing individuals to regulate walking speed in real time via adaptive control mechanisms (Song et al., 2020; Downer et al., 2023). Despite the widespread adoption of dual-task gait paradigms, there remains limited consensus on baseline dual-task behaviour under self-regulated locomotion, particularly when cognitive load is systematically modulated. This lack of methodological consensus complicates interpretation of exaggerated dual-task effects reported in clinical populations, as it remains unclear to what extent such effects reflect pathology versus task-induced artefacts. Previous work has demonstrated that self-paced treadmills preserve key features of natural gait and yield reliable spatiotemporal measures that closely resemble overground walking (Sloot et al., 2014a; Sloot et al., 2014b). This characteristic is particularly relevant for dual-task paradigms, where externally imposed pacing may inflate or obscure cognitive–motor interference effects (Al-Juaid and Al-Amri, 2020; Kao and Pierro, 2021). When combined with virtual reality (VR), self-paced treadmills also allow the presentation of ecologically relevant visual contexts while maintaining experimental control.

In the present study, we introduced and evaluated a dual-task gait assessment protocol integrating VR-based self-paced treadmill walking and parametrically modulated N-back working memory tasks. The primary aim was not to test clinical hypotheses, but to establish proof of concept, as well as the stability and sensitivity of the protocol, in a healthy cohort, thereby providing a reference framework for subsequent application in patient populations with impaired cognitive–motor integration.

## Methods

### Study sample and experimental setting

Twenty-three healthy adult males (mean age: 34.56 ± 5.12; height: 171 ± 6 cm; weight: 77.22 ± 9.76; and BMI: 27.82 ± 6.93 kg/m^2^) underwent two gait analysis sessions separated by 5 ± 3 days. Eligibility criteria included age between 30 and 50 years, normal or corrected-to-normal vision, and no history of neurological, cardiovascular, or musculoskeletal conditions including epilepsy or balance impairments. All participants provided written informed consent prior to participation. The Cardiff University School of Healthcare Science Research Ethics Committee approved the study.

Data were collected in Research Centre for Clinical Kinesiology at Cardiff University using Gait Real- time Analysis Interactive Lab (GRAIL, Motek). The system comprises a dual-belt instrumented treadmill with self-paced walking capability, embedded force plates, a 180° curved virtual reality projection screen, and a 12-camera VICON MX optical tracking system (Oxford Metrics, UK) sampling at 100 Hz. This setup allows precise measurement of gait parameters in a controlled, immersive environment. The virtual environment was delivered using a large-field, non-immersive projection system (180° curved screen) integrated within the GRAIL. Visual flow speed was continuously synchronised to each participant’s self-paced treadmill velocity to preserve the coupling between locomotor output and optic-flow input. Participants did not wear a head-mounted display and no explicit interaction with virtual objects was required during walking.

### Procedure

Each participant completed two sessions approximately one week apart to evaluate data reliability. In each session, participants walked under three randomised conditions: single-task (ST) walking at a self-selected pace, and dual-task (DT) walking combined with auditory 1-back and 2-back working memory tasks.

Twenty-five reflective markers were attached to anatomical landmarks of the lower limbs using the Human-Body-Model lower-body marker set, enabling detailed kinematic data collection. Under dual-task conditions, participants listened to a sequence of auditory digits and responded by pressing a handheld button when the current digit matched either the immediately preceding digit (1-back) or the digit two positions earlier (2-back). Participants underwent a 6-min familiarisation period walking at a self-selected pace to acclimate to both treadmill and VR environment.

At the beginning of each trial, participants were instructed to stand centrally on the split-belt treadmill, which incorporates two embedded force plates, with each foot positioned on a separate plate. The treadmill belts were set to identical speeds matching each participant’s self-paced walking velocity. The speed of the visual flow in the virtual reality environment was continuously synchronized with the participant’s walking speed to maintain ecological validity, as described by Al-Amri et al. (2017). Each session began with a familiarization period in which the participants walked at their comfortable pace for a minimum of six minutes. Following familiarization, participants completed three six-minute walking trials on the GRAIL treadmill under the following conditions: free walking (single-task), dual-task with 1-back cognitive load, and dual-task with 2-back cognitive load. The order of these trials was randomized for each participant to minimize potential learning or order effects. Two-minute seated rest intervals were provided between trials to reduce the risk of fatigue.

### Data processing

Motion capture data were filtered using a second-order Butterworth low-pass filter with a cut off frequency of 10 Hz. Gait events were detected using foot markers data, while gait speed was derived from treadmill output. Spatiotemporal parameters, including step width, step length, stride time, cadence, and stance time, were processed in MATLAB (The MathWorks, Inc.). Gait Stability Ratio (GSR) was calculated as the ratio of cadence to gait speed to estimate stability (Cromwell and Newton, 2004). To minimise the influence of gait initiation and termination, analyses focused on 100 consecutive strides beginning 50 s into each trial.

### Statistical analysis

All analyses were conducted in MATLAB (R2024b; The MathWorks, Inc.) using custom scripts. Tests were two-tailed with *α* = 0.05. Normality of paired difference scores was assessed. Effects of task condition (single-task, 1-Back, 2-Back) and session were examined using two-way repeated-measure ANOVA. Where required, planned pairwise comparisons were performed using paired t-tests. Dual-task cost (DTC) for each gait parameter was calculated as percentage change from single- to dual-task performance:


$$\mathrm{DTC}(\%)=\frac{\left(\text{Dual task value}-\text{single task value}\right)}{\text{single task value}}*100\%$$

DTC was compared between sessions and between 1-Back and 2-Back conditions using paired tests consistent with distributional assumptions. Cognitive-task performance was summarised using N-back response accuracy; reaction times were not recorded, therefore, cognitive performance is reported as accuracy only.

Test–retest reliability was estimated using a two-way mixed-effects, absolute-agreement, single-measure intraclass correlation coefficient (ICC (A,1)), appropriate for two fixed study sessions. Ninety-five percent confidence intervals for ICC were obtained from ANOVA mean squares using F-distribution–based methods. Measurement precision was summarised using the standard error of measurement (SEM) and minimal detectable change at 95% confidence (MDC_95_), computed using standard definitions. Relative variability was described using coefficient of variation (CV%) (pooled SD divided by grand mean). Agreement was evaluated using Bland–Altman mean bias and 95% limits of agreement, and proportional bias was assessed by regressing session differences on participant means.

## Results

**Figure 1:**
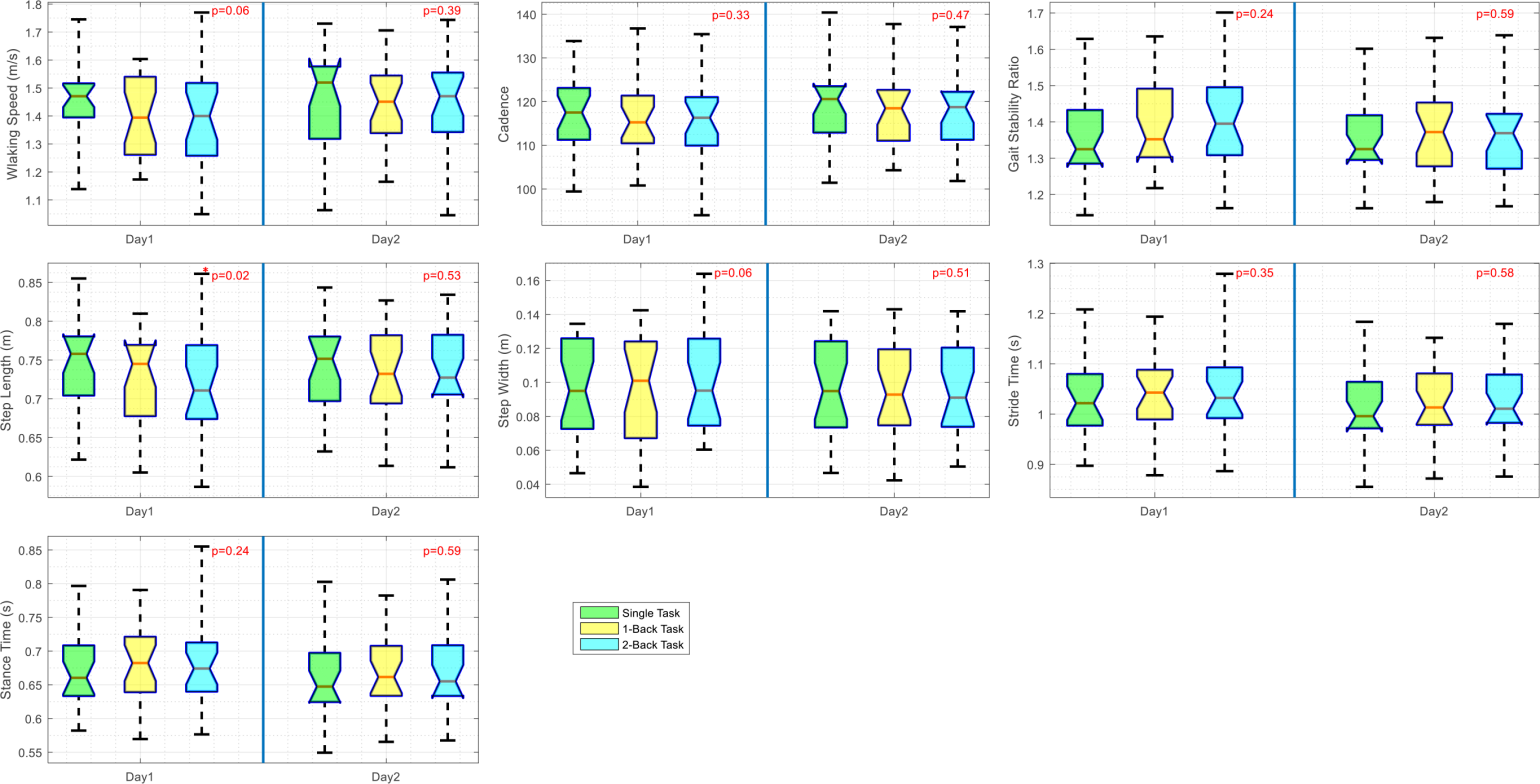
Changes in selected spatiotemporal parameters across single-task (ST) and dual-task (DT) conditions.

Twenty-three participants completed both testing sessions. Median values and standard deviations for spatiotemporal gait parameters across task conditions and sessions are presented in Figure 1. No significant differences were observed within or between sessions for most parameters (*p* ≥ 0.05), with the exception of step length during the first session (*p* = 0.02). Gait speed and step length were consistently higher during single-task walking than during dual-task conditions, with progressive reductions from single-task to 1-back and 2-back conditions. Stride time was shortest in the single-task condition across both sessions. Step length in Day 1 differed significantly between single-task and N-back dual-task conditions, indicating a cognitive load–related alteration in gait pattern.

**Figure 2:**
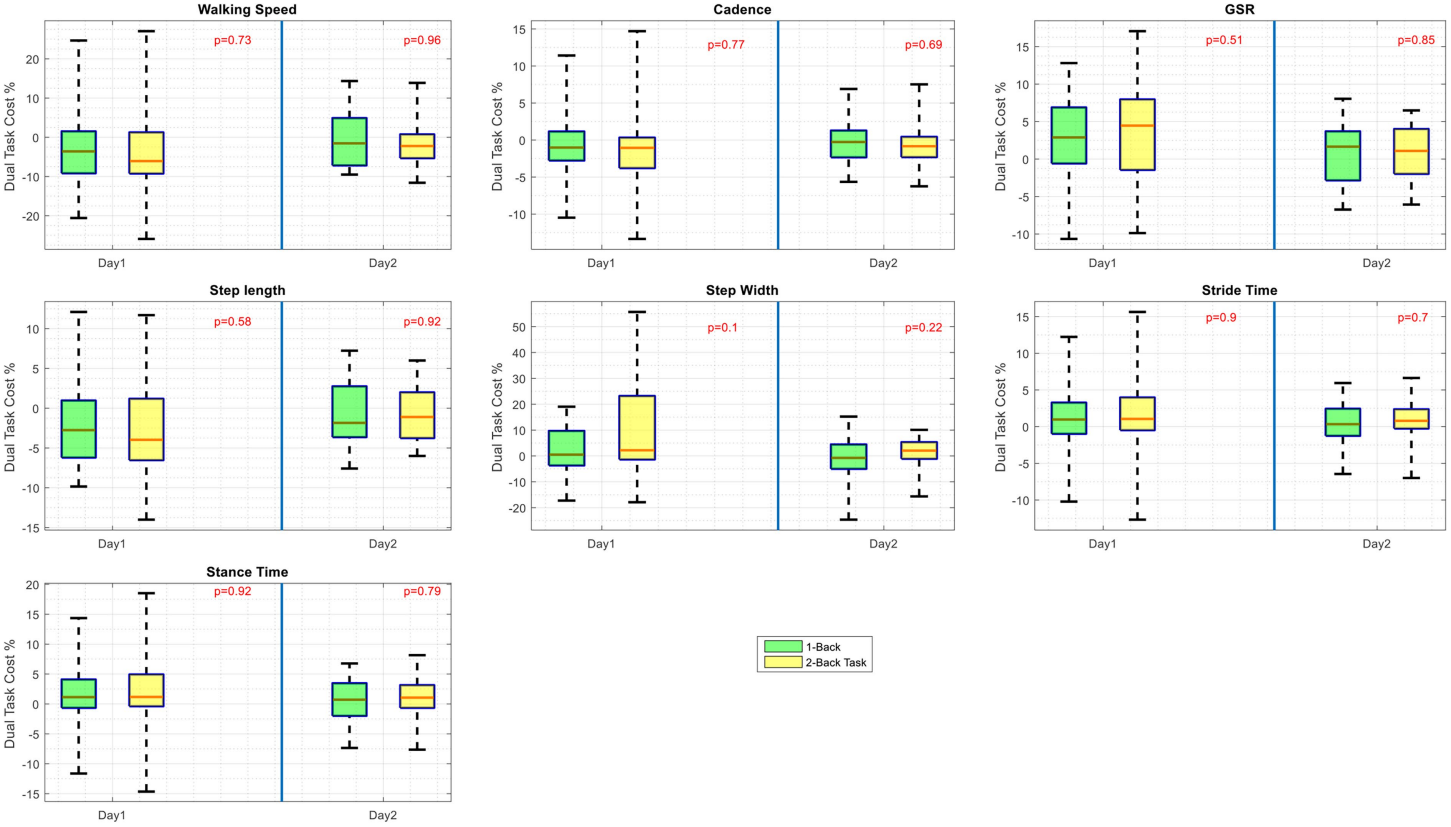
Dual task costs of selected gait parameters.

DTC for gait parameters are illustrated in Figure 2. No significant differences in DTC were found between sessions or between the two N-back difficulty levels. DTC values remained statistically unchanged across sessions and cognitive load levels. A trend toward reduced DTC was observed for step width and stride time across sessions, suggesting potential participant adaptation to the dual-task protocol over time.

**Figure 3:**
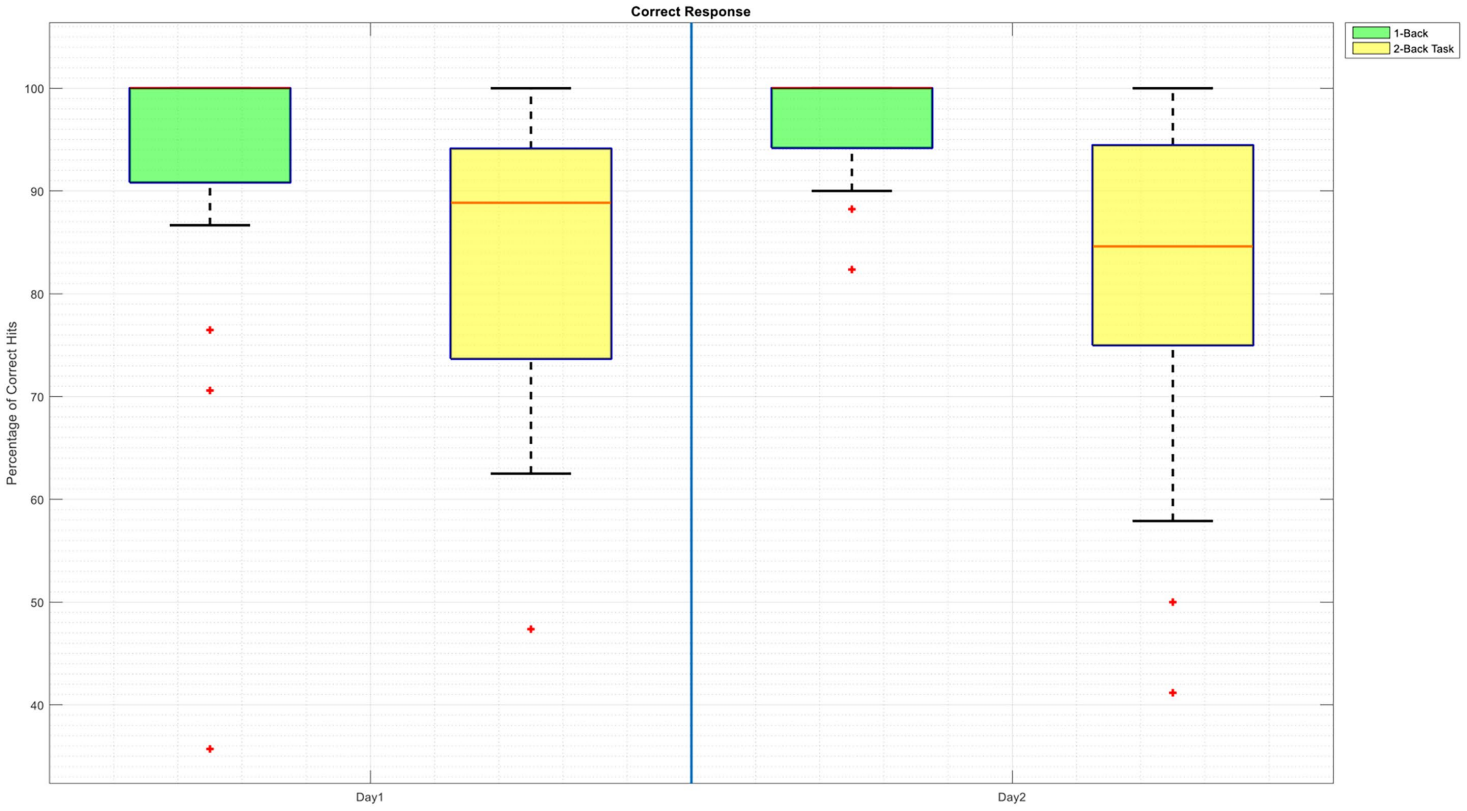
N-back response accuracy plot for different difficulty levels.

Figure 3 displays N-back test accuracy. Participants performed less accurately on the 2-back condition compared to the 1-back condition, consistent with the increased cognitive demands imposed by higher load. This pattern confirms that the 2-back condition imposed greater cognitive demand than the 1-back condition.

### Test–retest reliability

#### Average gait metrics

Test–retest reliability of mean spatiotemporal gait parameters ranged from moderate to excellent across all three walking conditions (Table 1). Walking speed demonstrated good reliability during Free Walking (ICC = 0.767, 95%CI: 0.527–0.894, SEM = 0.078 m/s, MDC95 = 0.217 m/s), with slightly lower but acceptable reliability during the 1-Back (ICC = 0.675, 95%CI: 0.373–0.847) and 2-Back (ICC = 0.682, 95%CI: 0.384–0.851) tasks. Changes of approximately 0.22–0.27 m/s would be required to exceed measurement error and reflect true change in walking speed.

**Table 1:** Test–retest reliability and effect sizes for spatiotemporal gait parameters across walking conditions.

Gait parameter	Condition	Mean D1 (±SD)	Mean D2 (±SD)	ICC (95% CI)	SEM	MDC95	BA Bias	LoA	Cohen’s *d*	η^2^
Average metrics										
Walking speed (m/s)	Free walking	1.448 (0.153)	1.467 (0.171)	0.767 (0.527–0.894)	0.078	0.217	−0.019	−0.236, 0.198	−0.17	0.003
	1-Back	1.392 (0.145)	1.444 (0.141)	0.675 (0.373–0.847)	0.082	0.226	−0.052	−0.278, 0.175	−0.45	0.033
	2-Back	1.384 (0.189)	1.445 (0.160)	0.682 (0.384–0.851)	0.099	0.274	−0.061	−0.335, 0.213	−0.44	0.031
Gait stability ratio	Free walking	1.359 (0.111)	1.358 (0.114)	0.786 (0.560–0.903)	0.052	0.144	0.001	−0.144, 0.145	0.01	0.000
	1-Back	1.400 (0.121)	1.370 (0.110)	0.770 (0.532–0.895)	0.056	0.154	0.030	−0.124, 0.184	0.38	0.017
	2-Back	1.412 (0.151)	1.369 (0.122)	0.760 (0.515–0.890)	0.067	0.187	0.042	−0.144, 0.229	0.45	0.024
Cadence (steps/min)	Free walking	117.18 (7.65)	118.51 (8.75)	0.902 (0.785–0.957)	2.565	7.110	−1.332	−8.442, 5.777	−0.37	0.007
	1-Back	115.97 (7.66)	117.94 (7.66)	0.860 (0.700–0.938)	2.862	7.932	−1.968	−9.901, 5.964	−0.49	0.017
	2-Back	115.65 (8.24)	117.71 (7.84)	0.770 (0.532–0.895)	3.856	10.688	−2.068	−12.756, 8.621	−0.38	0.017
Step width (m)	Free walking	0.096 (0.030)	0.096 (0.027)	0.852 (0.684–0.934)	0.011	0.031	−0.001	−0.031, 0.030	−0.06	0.000
	1-Back	0.097 (0.031)	0.095 (0.028)	0.871 (0.721–0.943)	0.011	0.030	0.002	−0.028, 0.032	0.13	0.001
	2-Back	0.101 (0.031)	0.097 (0.027)	0.850 (0.680–0.933)	0.011	0.031	0.004	−0.027, 0.036	0.27	0.006
Step length (m)	Free walking	0.741 (0.056)	0.742 (0.057)	0.786 (0.561–0.903)	0.026	0.073	0.000	−0.073, 0.072	−0.01	0.000
	1-Back	0.722 (0.060)	0.736 (0.058)	0.773 (0.536–0.897)	0.028	0.078	−0.013	−0.091, 0.064	−0.33	0.013
	2-Back	0.719 (0.072)	0.737 (0.060)	0.766 (0.526–0.894)	0.032	0.089	−0.018	−0.107, 0.071	−0.40	0.019
Stance time (s)	Free walking	0.670 (0.050)	0.662 (0.059)	0.875 (0.729–0.945)	0.019	0.053	0.007	−0.046, 0.061	0.27	0.005
	1-Back	0.682 (0.050)	0.666 (0.050)	0.740 (0.480–0.881)	0.026	0.071	0.016	−0.055, 0.087	0.45	0.027
	2-Back	0.683 (0.059)	0.667 (0.052)	0.697 (0.408–0.859)	0.031	0.085	0.016	−0.069, 0.101	0.36	0.020
Stride time (s)	Free walking	1.030 (0.069)	1.018 (0.076)	0.897 (0.774–0.955)	0.023	0.064	0.011	−0.053, 0.075	0.34	0.006
	1-Back	1.043 (0.069)	1.022 (0.066)	0.791 (0.569–0.906)	0.031	0.086	0.021	−0.065, 0.106	0.47	0.024
	2-Back	1.044 (0.078)	1.025 (0.068)	0.711 (0.432–0.866)	0.039	0.109	0.019	−0.089, 0.128	0.35	0.018
Variability metrics										
Walking speed var.	Free walking	0.129 (0.019)	0.128 (0.020)	0.613 (0.278–0.815)	0.012	0.033	0.001	−0.032, 0.034	0.03	0.000
	1-Back	0.135 (0.029)	0.127 (0.018)	0.520 (0.148–0.763)	0.017	0.046	0.008	−0.038, 0.054	0.34	0.028
	2-Back	0.130 (0.020)	0.130 (0.024)	0.870 (0.719–0.943)	0.008	0.022	0.000	−0.022, 0.022	0.02	0.000
GSR variability	Free walking	0.039 (0.020)	0.033 (0.013)	0.305 (−0.112–0.631)	0.014	0.038	0.006	−0.032, 0.045	0.31	0.034
	1-Back	0.059 (0.051)	0.032 (0.015)	−0.003 (−0.406–0.400)	0.038	0.105	0.026	−0.079, 0.131	0.49	0.111
	2-Back	0.043 (0.021)	0.033 (0.015)	0.275 (−0.144–0.611)	0.016	0.043	0.010	−0.034, 0.053	0.44	0.067
Cadence variability	Free walking	9.926 (1.412)	9.736 (1.072)	0.688 (0.394–0.854)	0.700	1.941	0.190	−1.751, 2.131	0.19	0.006
	1-Back	10.787 (3.509)	9.820 (1.159)	0.131 (−0.287–0.508)	2.436	6.751	0.967	−5.784, 7.718	0.28	0.035
	2-Back	9.924 (1.224)	9.831 (1.154)	0.861 (0.701–0.938)	0.443	1.229	0.093	−1.136, 1.323	0.15	0.002
Step width var. (m)	Free walking	0.020 (0.005)	0.020 (0.004)	0.834 (0.648–0.926)	0.002	0.005	0.001	−0.004, 0.006	0.25	0.005
	1-Back	0.020 (0.005)	0.020 (0.005)	0.750 (0.497–0.885)	0.003	0.007	0.000	−0.007, 0.007	0.05	0.000
	2-Back	0.020 (0.006)	0.020 (0.006)	0.906 (0.792–0.959)	0.002	0.005	0.000	−0.005, 0.005	0.05	0.000
Step length var. (m)	Free walking	0.022 (0.008)	0.019 (0.006)	0.427 (0.029–0.709)	0.005	0.014	0.003	−0.011, 0.018	0.43	0.052
	1-Back	0.030 (0.025)	0.018 (0.006)	0.150 (−0.269–0.522)	0.017	0.047	0.013	−0.034, 0.059	0.53	0.110
	2-Back	0.022 (0.008)	0.019 (0.009)	0.327 (−0.088–0.645)	0.007	0.019	0.003	−0.015, 0.022	0.34	0.039
Stance time var. (s)	Free walking	0.019 (0.017)	0.014 (0.006)	0.218 (−0.203–0.571)	0.011	0.031	0.005	−0.026, 0.036	0.30	0.036
	1-Back	0.041 (0.085)	0.014 (0.006)	−0.016 (−0.416–0.390)	0.061	0.168	0.027	−0.142, 0.195	0.31	0.049
	2-Back	0.018 (0.008)	0.015 (0.007)	0.497 (0.116–0.750)	0.005	0.014	0.002	−0.012, 0.017	0.34	0.030
Stride time var. (s)	Free walking	0.021 (0.016)	0.017 (0.008)	0.276 (−0.143–0.611)	0.011	0.029	0.005	−0.025, 0.034	0.30	0.034
	1-Back	0.045 (0.083)	0.017 (0.008)	−0.018 (−0.418–0.388)	0.060	0.166	0.028	−0.138, 0.194	0.33	0.056
	2-Back	0.021 (0.010)	0.018 (0.009)	0.558 (0.199–0.785)	0.006	0.017	0.003	−0.015, 0.020	0.30	0.020

ICC = Intraclass Correlation Coefficient; 95%CI = 95% Confidence Interval; SEM = Standard Error of Measurement; MDC95 = Minimal Detectable Change at 95% confidence; BA Bias = Bland–Altman mean difference; LoA = 95% Limits of Agreement; Cohen’s d = paired effect size between Day 1 and Day 2; η^2^ = eta-squared. ICC benchmarks: <0.50 Poor; 0.50–0.75 Moderate; 0.75–0.90 Good; >0.90 Excellent.

GSR showed good reliability across all conditions (Free Walking: ICC = 0.786; 1-Back: ICC = 0.770; 2-Back: ICC = 0.760), along with small SEM values (0.052–0.067) and MDC95 of 0.144–0.187, which confirms GSR as a stable and reliable measure of gait stability under both single-task and dual-task walking. Cadence exhibited the highest reliability among average gait metrics, ranging from good to excellent. Reliability was highest during Free Walking (ICC = 0.902, 95%CI: 0.785–0.957) and reduced progressively through 1-Back (ICC = 0.860) and 2-Back (ICC = 0.770) conditions. MDC95 for cadence ranged from 7.1 to 10.7 steps/min.

Spatial parameters remained consistently reliable regardless of cognitive load. Step width ICC ranged from 0.850 to 0.871 with an SEM of 0.011 m and MDC95 of approximately 0.030–0.031 m. Step length ICC ranged from 0.766 to 0.786 with SEM values of 0.026–0.032 m and MDC95 of 0.073–0.089 m. On the other hand, temporal gait parameters demonstrated a systematic decline in reliability with increasing cognitive load. Stance time reliability was good during Free Walking (ICC = 0.875, 95%CI: 0.729–0.945) but reduced to moderate during 1-Back (ICC = 0.740) and 2-Back (ICC = 0.697) conditions. Stride time followed the same pattern, from excellent during Free Walking (ICC = 0.897, 95%CI: 0.774–0.955) to moderate during the 2-Back condition (ICC = 0.711). Bland–Altman analysis revealed small systematic bias for all average gait metrics, with mean differences close to zero across conditions, indicating no consistent directional error between sessions. Cohen’s d was small to negligible across all average metrics (range: −0.057 to −0.486), with eta-squared values below 0.033, confirming that between-session differences explained a negligible proportion of total variance.

#### Gait variability metrics

Gait variability metrics exhibited lower and more variable reliability compared to average metrics (Table 1). Step width variability was the most reliable variability measure, showing good to excellent reliability across all conditions (Free Walking: ICC = 0.834; 1-Back: ICC = 0.750; 2-Back: ICC = 0.906), with very small SEM (0.002–0.003 m) and MDC95 (0.005–0.007 m). Variability in walking speed showed moderate reliability during Free Walking (ICC = 0.613) and 1-Back (ICC = 0.520), but good reliability during 2-Back (ICC = 0.870). Cadence variability was moderately reliable during Free Walking (ICC = 0.688) and good in the 2-Back condition (ICC = 0.861), but showed poor reliability during 1-Back (ICC = 0.131, SEM = 2.436, MDC95 = 6.751 steps/min).

GSR variability showed poor reliability across all conditions (ICC: −0.003 to 0.305). In particular, the 1-Back condition approached zero reliability (ICC = −0.003, 95%CI: −0.406–0.400), indicating that between-session variance exceeded between-subject variance. Step length variability ranged from poor to moderate reliability (ICC: 0.150–0.427), particularly during 1-Back (ICC = 0.150). Stance time and stride time variability showed consistently poor reliability across conditions, with ICC values ranging from −0.016 to 0.497 and −0.018 to 0.558 respectively, and large MDC95 values (up to 0.168 s) that exceeded the mean values themselves in several conditions.

## Discussion

This study examined the reliability and baseline gait performance associated with a dual-task paradigm integrating VR-based self-paced treadmill walking and graded working memory load. Across sessions and task conditions, spatiotemporal gait parameters were largely stable in healthy adults. Collectively these findings indicate that the primary contribution of this work lies in establishing a robust, ecologically grounded methodological framework suitable for future clinical investigation, rather than in detecting large dual-task effects in neurologically intact participants.

Spatiotemporal gait parameters showed moderate to excellent test–retest reliability across conditions, with walking speed, gait stability ratio, and cadence particularly stable. Temporal measures showed slight decreases under higher cognitive load. Low measurement error and minimal detectable change indicate that small differences reflect true changes, providing a reliable baseline for future cognitive–motor studies. On the other hand, Gait variability metrics showed reduced and less consistent reliability compared to average gait parameters. Step width variability was the only measure with consistently good reliability. While temporal measures exhibited poor reliability and high measurement error. Reliability improved under higher cognitive load, suggesting attentional demand may constrain motor variability. These findings highlight the task-dependent nature of variability metrics and warrant cautious interpretation in healthy populations.

The absence of statistically significant dual-task effects across most gait parameters is consistent with contemporary evidence in healthy cohorts, in which cognitive–motor interactions typically manifest as subtle, adaptive modulations rather than overt interference. Step length decreased significantly under dual-task conditions, with a further reduction observed as N-back difficulty increased. Other gait parameters showed consistent directional trends without reaching statistical significance, suggesting a shift toward a more cautious and stabilising gait strategy under cognitive demand. These adaptations align with the “posture second” strategy, whereby healthy adults prioritise cognitive performance over gait efficiency during dual tasking (Kelly et al., 2013). Nevertheless, inter-individual variability may have caused the relatively small and non-significant effects observed in other gait parameters as dual task prioritisation can vary between individuals.

The magnitude of dual-task interference observed here was smaller than in several previous studies (Kao and Pierro, 2021; Hollman et al., 2006; McPhee et al., 2022), potentially due to methodological factors. First, the use of a self-paced treadmill allows participants to dynamically regulate walking speed, preserving more natural locomotor patterns compared with fixed-speed treadmills (Sloot et al., 2014a). This flexibility may enable compensatory strategies that minimise the observable impact of cognitive load. Second, the VR environment provides continuous, meaningful visual input, supporting visuomotor integration and sustained attentional engage (De Keersmaecker et al., 2023). Third, the cohort comprised healthy, middle-aged males with no known neurological or cognitive impairments, likely possessing sufficient cognitive reserve and neuromuscular coordination to manage dual task demands. Finally, the standardised familiarisation period and randomised trial order has likely reduced variability from learning or fatigue effects.

The inclusion of the N-back paradigm was a deliberate methodological choice. As a validated and widely used measure of working memory and executive function, the N-back task enables systematic manipulation of cognitive load (Meule, 2017). The observed decline in response accuracy in the two-back condition confirms that participants experienced increasing cognitive demand. Concurrent gait adaptations, most clearly observed in step length, support the sensitivity of this dual-task framework for capturing real-time cognitive–motor interactions in healthy adults. These findings are relevant for clinical translation, where such interactions may indicate early functional decline or emerging neurological dysfunction, especially in populations such as individuals with traumatic brain injury, stroke survivors, or patients with Parkinson’s disease.

Despite these strengths, several limitations warrant consideration. First, the cohort comprised healthy middle-aged males only, limiting generalisability; sex-, age-, and fitness-related differences in spatiotemporal gait, gait variability, and dual-task prioritisation strategies may yield different interference patterns and reliability estimates in more representative samples. Second, the modest sample size, typical of feasibility and reliability work, reduces power to detect small dual-task effects and results in wider confidence intervals for some reliability metrics; therefore, null hypothesis-test findings should not be interpreted as evidence of equivalence. Third, walking was performed on a self-paced treadmill within a projection-based, non-immersive VR environment; although this improves experimental control, findings may not fully generalise to overground walking or immersive head-mounted VR, which can impose different sensory conflicts and attentional demands. Fourth, cognitive performance was summarised using N-back accuracy only because reaction times were not recorded, limiting the granularity of cognitive–motor inference. Finally, although familiarisation and rest periods were used, unmeasured influences of learning or adaptation, cognitive or physical fatigue, and individual task-prioritisation could contribute to inter-individual variability and modest dual-task costs; future studies should quantify these factors explicitly and refine VR scenarios and cognitive task parameters to further enhance ecological validity and clinical sensitivity.

Gait in natural environments requires continuous integration of multisensory information alongside cognitive processes such as navigation and task prioritisation. Paradigms simulating supermarket navigation (Kim et al., 2015) or road crossing (Nieborowska et al., 2019) are particularly valuable for assessing readiness for community reintegration in clinical cohorts. For example, dual-task assessments in individuals with mild traumatic brain injury have revealed subtle impairments that may not appear in standard clinical assessments (Howell et al., 2018). Identifying such deficits is critical for accurately monitoring post-injury recovery, informing rehabilitation targets, and managing patient and family expectations.

Taken together, these findings indicate that cognitive loading interacts with gait control by producing modest, systematic adaptations, significant in step length and trending in other parameters, rather than disruptive interference. This supports the suitability of the paradigm for probing more pronounced deficits in clinical populations. Treadmill-based gait analysis remains popular due to efficiency, controlled environment, and capacity to capture multiple gait parameters simultaneously. Familiarisation is essential to reduce treadmill-induced gait effects (Arnold et al., 2019; Oude Lansink et al., 2017; Schellenbach et al., 2010; Zeni and Higginson, 2010; Collins et al., 2013). In this study, a 6-min familiarisation period was employed, excluding the first and final minutes of testing, yielding consistent performance across sessions. The absence of practice effects indicates that task novelty was minimised, providing a reliable baseline for future comparisons with clinical cohorts. The N-back task was chosen for its versatility and established sensitivity to working memory. Previous studies have identified neural correlates using neuroimaging (Dores et al., 2017; Liu et al., 2017; Sala-Llonch et al., 2012), demonstrated convergence with established neuropsychological assessments (Miller et al., 2009), and reported successful application in pathological populations including traumatic brain injury, stroke, and Parkinson’s disease (Demeter et al., 2017; Owens et al., 2018). Accordingly, the present framework is well suited for extension to neuroimaging studies and application across diverse patient groups in future research.

Advances in VR technology have informed rehabilitation strategies by improving ecological fidelity, safety, and participant engagement. Virtual environments allow individuals to practise potentially hazardous but routine tasks in controlled settings (Nieborowska et al., 2019), promote sustained motivation, and facilitate the development of individualised rehabilitation programmes. Emerging evidence supports the use of VR-based dual-task paradigms for both assessment and intervention across clinical populations. For instance, executive function has been assessed in veterans with mild traumatic brain injury using VR-based dual-task games, while stroke survivors have benefited from VR-based evaluations of visuospatial neglect and collision risk during walking (Aravind and Lamontagne, 2017). Furthermore, randomised controlled trials have demonstrated improvements in gait parameters following VR-based dual-task interventions in individuals with chronic stroke. The present protocol can be feasibly adapted to support such VR-based assessments and rehabilitation interventions, reinforcing its translational potential.

## Conclusion

This study establishes a reliable, ecologically grounded VR-based dual-task paradigm for the simultaneous assessment of gait and working memory during self-paced walking. In healthy adults, the framework demonstrated stable spatiotemporal gait behaviour across sessions, with modest, load-dependent adaptations, significant for step length and trending for other parameters, consistent with flexible cognitive–motor regulation rather than overt interference. These findings confirm the paradigm as a robust methodological baseline for evaluating pathological gait and cognitive–motor dysfunction. Future work should extend this approach to clinical populations and incorporate neuroimaging to further elucidate the neural mechanisms underlying dual-task performance and its disruption in neurological conditions.

## Data Availability

The raw data supporting the conclusions of this article will be made available by the authors upon reasonable request, subject to ethical approval requirements, participant confidentiality, and applicable data protection regulations.
